# Accession-Level Differentiation of Urushiol Levels, and Identification of Cardanols in Nascent Emerged Poison Ivy Seedlings

**DOI:** 10.3390/molecules24234213

**Published:** 2019-11-20

**Authors:** Aneirin A. Lott, Emily R. Baklajian, Christopher C. Dickinson, Eva Collakova, John G. Jelesko

**Affiliations:** 1 School of Plant and Environmental Science, Virginia Tech, 220 Ag Quad Lane, Blacksburg, VA 24061, USA; 2 Plant Molecular and Cellular Biology, University of Florida, 255 Hull Road, Fifield Hall, Gainesville, FL 32611-0690, USA

**Keywords:** poison ivy, *Toxicodendron radicans*, urushiol, cardanol, and cardanol hydroxylase

## Abstract

Poison ivy (*Toxicodendron radicans* (L.) Kuntze) shows accession-level differentiation in a variety of morphometric traits, suggesting local adaptation. To investigate whether the presumed defense compound urushiol also demonstrates accession-level accumulation differences, in vitro nascent germinated poison ivy seedlings from geographically isolated populations were germinated in vitro and then assayed for known urushiol congener accumulation levels. Significant accession-level differences in the accumulation levels of total C15- and C17-, total C15-, total C17-, specific C15 congeners, and specific C17 congeners of urushiol were identified. In addition, hereto novel C15- and C17-urushiol isomers were identified as well. Cardanols are assumed to be the penultimate metabolites giving rise to urushiols, but this assumption was not previously empirically validated. C15-cardanol congeners and isomers corresponding to expected substrates needed to produce the observed C15-urushiol congeners and isomers were identified in the same poison ivy seedling extracts. Total C15-cardanol and C15-cardanol congeners also showed significant accession-level differences. Based on the observed C15-cardanol congeners in poison ivy, the penultimate step in urushiol biosynthesis was proposed to be a cardanol-specific hydroxylase activity.

## 1. Introduction

Poison ivy (*Toxicodendron radicans* (L) Kuntze) is a noxious native North American woody liana [1,2]. Poison ivy is widely known because human contact often results in “poison ivy rash” on exposed skin [3,4]. As many as 10–50 million cases of poison ivy rash occur each year [3,5]. The natural product produced by poison ivy that is responsible for causing the skin rash symptoms is generically called urushiol [6]. Urushiol is not a toxin, but rather a contact allergen. So, the skin rash symptoms are an immunological/allergenic response to urushiol-exposure on human skin [4]. The dermatological symptoms can last for 3–6 weeks and will eventually resolve into normal looking skin.

Urushiol is not a single compound, but rather a collection of closely related congeners. Urushiol is comprised of a 1,2-dihydroxy-3-alk(en)yl-benzene core structure. The alk(en)yl chain can be either a pentadec(en)yl or a heptadec(en)yl chain with zero to three vinyl bonds at conserved positions [7,8,9,10,11]. The ortho-1,2-dihydroxyl groups on urushiol are essential for allergenicity [6,12], and the severity of allergenicity increases with either a longer alkyl chain [13] and/or increasing number of double bonds [14]. Given these structural features both Dewick [15] and Giessman [16] proposed that urushiol is derived from fatty acid metabolism. Based on first principles, it was proposed that a fatty acid-CoA molecule is extended by a polyketide synthase activity into a tetraketide that subsequently undergoes sequential keto reduction, cyclization, and aromatization to give rise to anacardic acid. Anacardic acid is then presumed to be decarboxylated and hydroxylated to finally yield urushiol. Neither model differentiates whether the conversion of anacardic acid to urushiol occurs either as: two concerted-reactions with no stable intermediate (i.e., in a *nahG*-like fashion [17]), or in a two-step fashion first producing cardanol as a stable intermediate, which is subsequently hydroxylated into urushiol. None of the predicted metabolites from the tetraketide to cardanol have been identified in poison ivy extracts (or any other urushiol-producing plant species for that matter). With that said, poison ivy belongs to the family Anacardiaceae and other members of the Anacardiaceae produce related alkylphenol natural products. For example, cashew produces anacardic acid, cardanol, and cardol/alkylresorcinol [18], but not urushiol. Mango produces two other closely related alkylphenols: alkylresorcyclic acid and alkylresorcinol [19], but not urushiol.

Poison ivy is projected to respond favorably to projected increasing atmospheric CO_2_ accumulation levels. Poison ivy plants exposed to elevated CO_2_ levels grow faster, accumulate more biomass, and shift urushiol congener composition towards accumulating urushiols with higher degrees of unsaturation [20,21]. Urushiol congeners with more double bonds are allergenically more potent than congeners with fewer double bonds [14]. So, poison ivy is predicted to become not only more abundant but also more allergenic with the progression of the Anthropocene. Given these anticipated ecological impacts, there are enormous gaps in knowledge about both urushiol chemical ecology and poison ivy ecophysiology.

Poison ivy biogeography is well described from a taxonomic/systematics perspective. One overarching feature of poison ivy is the unusually high-degree of morphological variation observed both within and between poison ivy populations [1,2,22]. Nevertheless, *Toxicodendron radicans* (L) Kuntze shows consistent geographically-specific differentiation in a number of morphological traits sufficient to assign nine subspecies within the continental United States [2]. Such taxonomic-oriented studies focus on collected specimens and do not address whether the observed morphological traits are due to phenotypic plasticity or genetic differentiation between distant poison ivy populations. A common garden experiment utilizing poison ivy accessions from different locations demonstrated significant poison ivy accession-level differences for several biometric traits [23]. Thus, geographically-isolated poison ivy accessions seem to be genetically-differentiated at the level of biometric traits.

The present study focused on whether accession-level poison ivy differences include urushiol accumulation levels and congener composition at the earliest stage of poison ivy recruitment: nascent seedling emergence. Using in vitro seedling germination conditions, significant differences in urushiol accumulation levels of known urushiol congeners were observed between accessions, and novel isomers of urushiol congeners were identified. Cardanol congeners and isomers consistent with the expected immediate metabolic precursors for known urushiol congeners were also identified in this study. These findings support the hypothesis that C15-anacardic acid is converted to stable cardanol intermediates that are in turn the penultimate metabolites in urushiol biosynthesis.

## 2. Results

### 2.1. Accession-Level Differences in Steady State Total C15- and Total C17-Urushiol Accumulation Levels

To evaluate poison ivy accession-level urushiol levels, all seedlings were harvested at the same developmental stage. Scarified sterile drupes were placed in Magenta boxes [24] providing a homogeneous germination environment. Poison ivy germination showed a moderate to low degree of synchronous seedling emergence. Therefore, the seedlings were harvested at the nascent fully-emerged seedling stage (see methods), stored as frozen tissue, and alkylphenols were extracted analyzed by gas chromatography-mass spectrometry (GC-MS) [25].

**Figure 1:**
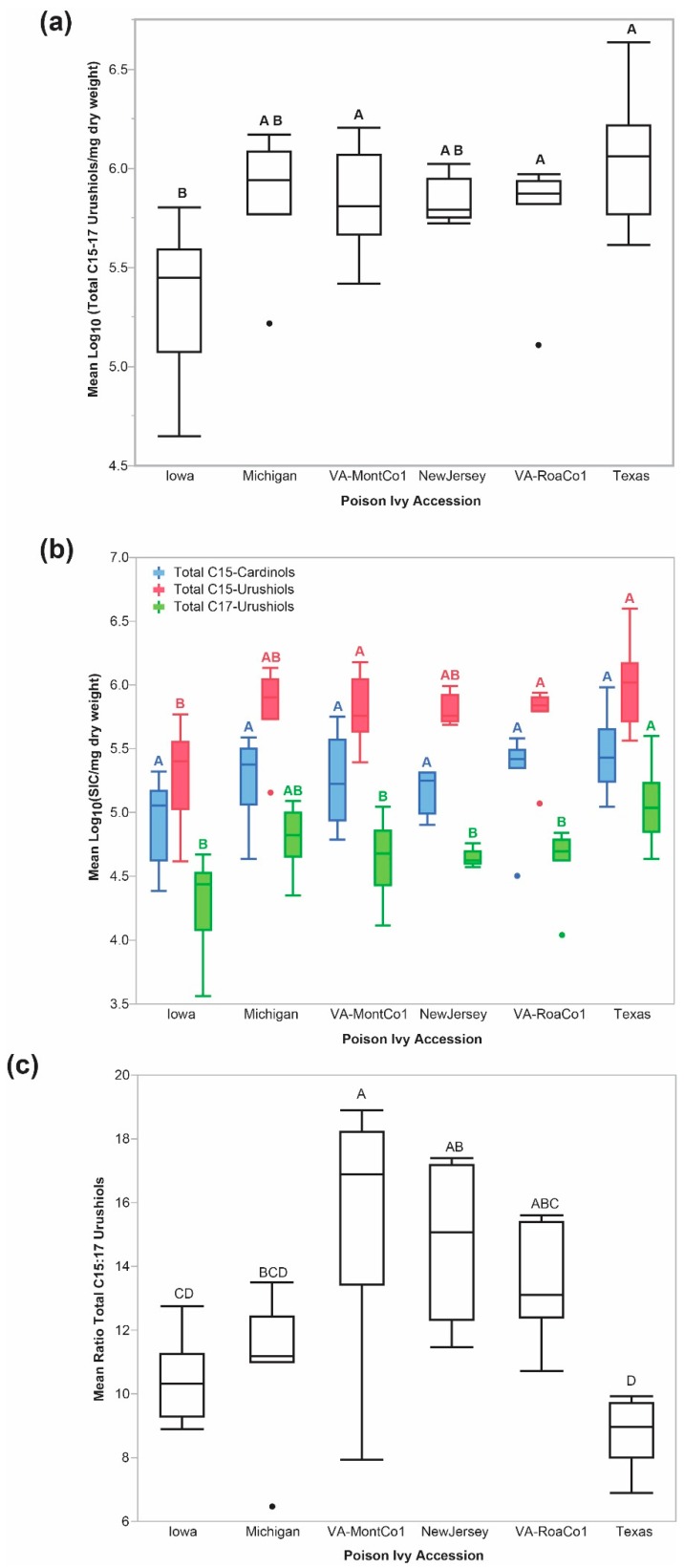
GC-MS analysis of alkylphenol levels in poison ivy accessions. (**a**) combined total C15- and C17-urushiol levels. (**b**) blue, total C15-cardanol; red, total C:15-urushiol; and green, total C17-urushiol levels. (**c**) ratio of total C15:C17 urushiol levels.

There were significant differences in steady state urushiol accumulation levels between poison ivy accessions collected from geographically isolated locations. Figure 1a illustrates that the steady state total urushiol accumulation levels (defined as combined total C15-urushiols and total C17-urushiols) in the Texas and the two Virginia accessions were significantly higher than those in the Iowa accession. These significant differences were due to larger steady state total C15-urushiol accumulation levels (Figure 1b) relative to lower the total C17-urushiol levels across all accessions. The ratio of total C15- to total C17-urushiols (total C15:C17-urushiol ratio) also showed significant accession-level differences with VA-MontCo1 significantly different from Iowa, Michigan, and Texas, as well as New Jersey significantly different than Iowa and Texas (Figure 1c). The maximum and minimum C15:C17 urushiol ratio values across all accessions differed by no more than 3-fold, suggesting that total C15- and total C17-urushiol levels in each seedling were highly correlated. Across all accessions, the range of total C15-urushiol levels between the 90% percentile and the 10% percentile was 7.7-fold. The most extreme differences (i.e., highest to lowest observed values) in total C15- and total C17-urushiol levels were both on the order of two orders of magnitude different (96.1-fold for total C15-urushiols, and 108.8-fold for total C17-urushiols). These most extreme values were not outliers because they approximated an expected normal quantile distribution for these Log_10_ transformed data.

### 2.2. Identification of Novel C15- and C17-Urushiol Isomers

**Figure 2:**
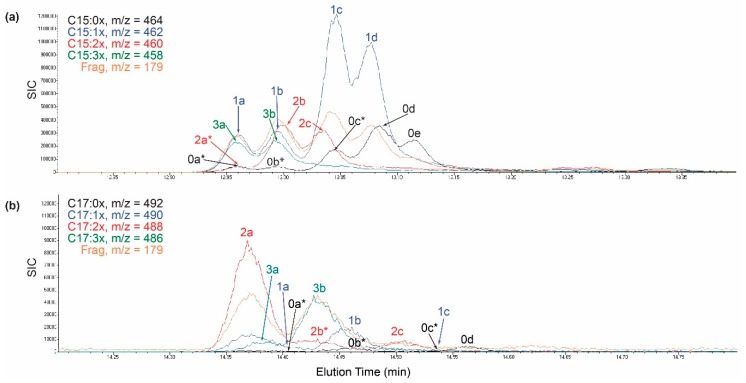
GC-MS selective ion chromatograms for C15- and C17-urushiol congeners and isomers. (**a**) sample C15-urushiol chromatogram. (**b**) sample C17-urushiol chromatogram. Numbers indicate the degree of unsaturation, and letters indicate unique isomer of the specified congener. Asterisk indicates apparent M+2 ion.

Urushiols are comprised of closely related ortho-dihydroxy-alk(en)yl-benzyne congeners that differ in both alkyl-chain length (15 carbons or 17 carbons) and degrees of unsaturation (0–3 double bonds at the Δ8, Δ10, and Δ12 positions). Previous reports using GC-MS of trimethyl-silane (TMS)-derivatized urushiols typically indicated a single chemical species for each urushiol congener, with the notable exceptions for two C15:1-urushiol isomers [26] and two C17:2 isomers [27]. This study of nascent fully-emerged poison ivy seedlings showed an expanded collection of C15-urushiol isomers with identical *m*/*z* ratios, but different elution times (Figure 2a). Urushiol isomers were labeled alphabetically according to their elution order, and apparent M+2 isotopes were additionally labeled with an asterisk. Some urushiol isomers were clearly M+2 isotopes with elution profiles matching a different urushiol with one additional degree of unsaturation. For example, Figure 2a illustrates that three C15:0 species (C15:0a*, C15:0b*, and C15:c*) were clearly M+2 isotopes of corresponding to co-eluting C15:1-urushiol isomers (C15:1a, C15:1b, and C15:1c, respectively). In addition to the three C15:1-urushiol isomers mentioned above, a fourth C15:1d-urushiol isomer was also identified. Two unique C15:0-urushiol congeners (C15:0d, and C15:0e) were observed. Two C15:2-urushiol isomers (C15:2b and C15:2c), and two C15:3-urushiol isomers (C15:3a and C15:3b) were also identified (Figure 2a, and Supplemental Figure S1a).

New isomers of C17-urushiols were also observed. Figure 2b shows there was only one unique C17:0-congener (C17:0d). In contrast, taken together Figure 2b and Figure S1b,c demonstrated two C17:2-urushiol isomers (C17:2a and C17:2c), two C17:3-urushiol isomers (C17:3a and C17:3b), and three C17:1-urushiol isomers (C17:1a, C17:1b, and C17:1c). Given the much lower C17-urushiol levels relative to the more abundant C15-urushiol levels (Figure 1b), it was possible that additional C17-urushiol isomers may exist, but were below the limit of detection in this study. Urushiol congeners are exceedingly difficult to completely isolate from one another at preparative scale from plant extracts. That, together with the overall small biomass of the newly emerged poison ivy seedlings, precluded detailed structural determination of each isomer.

**Figure 3:**
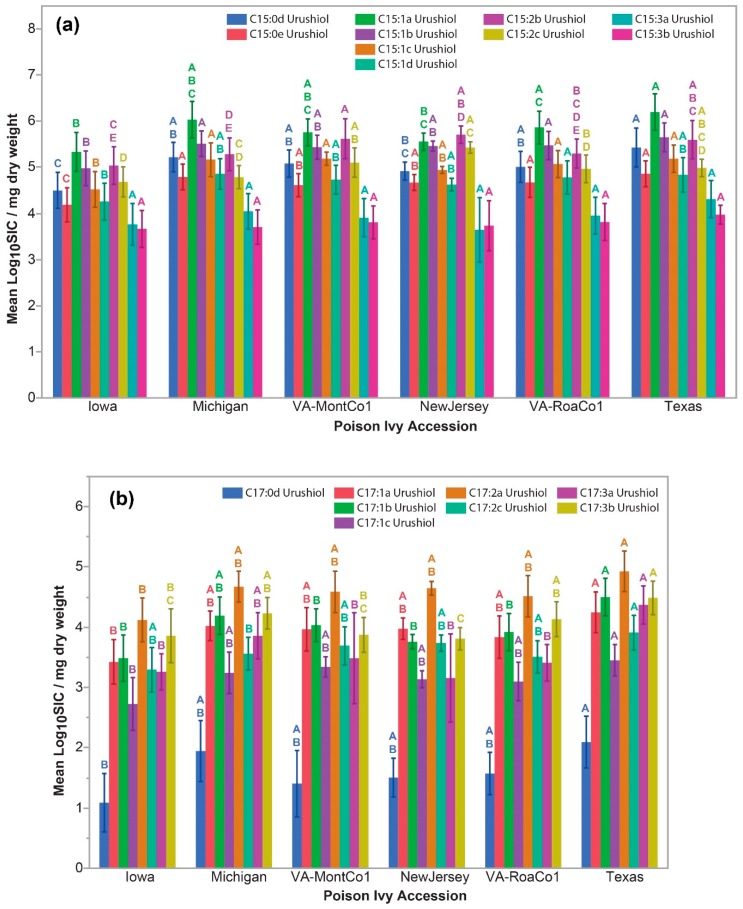
Steady state C15- and C17-urushiol congener and isomer accumulation levels. (**a**) C15-urushiols, (**b**) C17-urushiols. Error bars indicate standard deviation. Non-shared letters of the same color indicate statistically significantly different least square means between the poison ivy accessions.

The individual C15-urushiol and C17-urushiol isomers showed significant accession-level differences that generally, though not always, paralleled the accession-level differences observed with their respective total C15-urushiol and total C17-urushiol accumulation levels (Figure 3). The notable exceptions were the C15:3a- and C15:3b-urushiol isomers that accumulated to the lowest levels of all the C15-urushiol congeners, and did not show any significant differences in accumulation levels across the six accessions (Figure 3a). The C15-urushiol congeners showed a relative ordering of abundance levels with C15:1 ≥ C15:2 > C15:0 > C15:3. On the other hand, the C17-urushiol congeners showed a different relative ordering with C17:3 ≈ C17:2 ≥ C17:1 >> C17:0 (Figure 3b).

### 2.3. Urushiol Accumulation Imposed a Metabolic Load on Nascent Emerged Seedling Vigor

**Figure 4:**
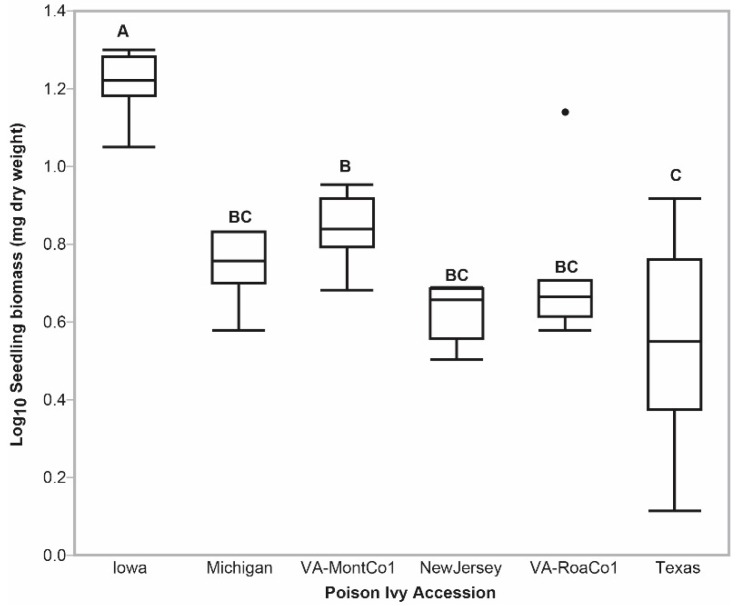
Poison ivy seedling biomass outlier box plots. Box plots that share at least one letter are not significantly different (*p*-value > 0.0500).

Three poison ivy accessions (Texas, VA-RoaCoa1, and VA-MontCo1) had significantly higher steady state total combined urushiols (Figure 1a) and total C15-urushiol (Figure 1b) accumulation levels compared to the Iowa accession. On the other hand, the Iowa accession had significantly larger total seedling dry biomass relative to the other five poison ivy accessions Figure 4. Thus, there was an inverse relationship between total dry seedling biomass and total urushiol accumulation levels. This pattern is consistent with total urushiol accumulation levels imposing a significant metabolic load on nascent emerged poison ivy seedlings.

### 2.4. Identification of C15-Cardanol Congeners and Isomers in Poison Ivy Seedlings

Cardanols are C15-alk(en)yl-phenols produced in other members of the Anacardiaceae family. C15-cardanol congeners isolated from cashew and derivatized with TMS produce parent and fragmentation ions with specific *m*/*z* ratios (Table 1, [18]). All TMS-derivatized cardanols produce congener-specific parent ions, congener-specific fragmentation ions, and a common and abundant *m*/*z* 180 fragmentation ion (Table 1).

**Figure 5:**
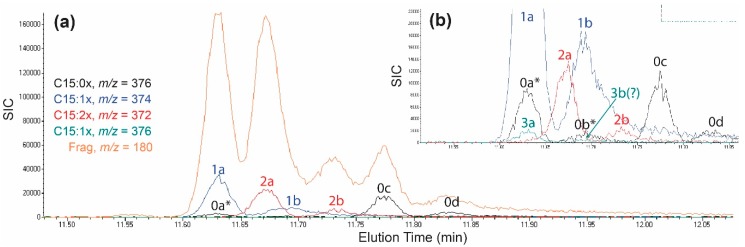
Selective ion chromatograms for C15-cardanol congeners and isomers. (**a**,**b**) Two C15-cardanol chromatograms illustrating different congener and isomers.

Figure 5a,b illustrates two nascent emerged poison ivy seedling GC-MS chromatograms that together illustrate the observed range of putative C15-cardanol congeners and their isomers. The C15:1a-, C15:0a*-, and C15:3a-cardanols coeluted with the same retention time. The C15:1b, C15:0b*, and C15:3b-cardanols likewise co-eluted. There was sufficient overlap between C15:1- and C15:2-cardanol parent ions that it was impractical to assign unambiguous C15:2 M+2 ions from C15:1-cardanols. The C15:0c- and C15:0d-cardanols were the last cardanol isomers to elute and were well separated from each other and the other cardanol congeners/isomers.

**Figure 6:**
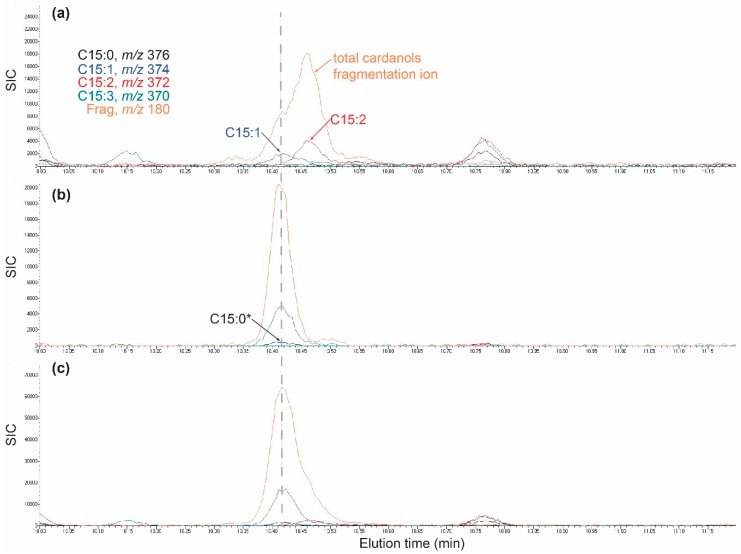
Selective ion chromatograms for C15-cardanols. (**a**) hairy root line HR32-4a. (**b**) 1 ng authentic 15:1-cardanol standard. (**c**) hairy root line HR32-4a extract spiked with 1 ng authentic C15:1-cardanol standard. Dotted vertical line indicates same retention time across all three chromatograms. Each panel has a different Y-axis scale.

Validation of at least one C15-cardanol was accomplished using extracts from a poison ivy hairy root line and an authentic C15:1 cardanol standard. An established poison ivy hairy root line HR32-4a displayed markedly reduced C15:2a-cardanol levels leaving an C15:1a-cardanol peak with an apparent C15:0a* [M+2] ion (Figure 6a). One nanogram of authentic C15:1-cardanol eluted at the same retention time as the putative C15:1a-cardanol *m*/*z* 374 ion from both the HR32-4a hairy root (Figure 6a,b) and the HR32-4a extract spiked with the authentic C15:1 cardanol standard (Figure 6c). The authentic C15:1-cardanol standard consistently showed a lower percentage of the parent *m*/*z* 374 ion relative to the most abundant fragmentation *m*/*z* 180 ion, compared to the published purified C15:1 cardanol isolated from cashew (Table 1). Nevertheless, the coeluting C15:1a cardanol ion in the HR32-4a extract showed *m*/*z* 374 to *m*/*z* 180 ion ratios that were in agreement with the authentic C15:1-cardanol standard (Table 1). Moreover, the apparent C15:0a* M+2 ion was not a genuine C15:0-cardanol ion because of a conspicuous deficit in an expected C15:0-cardanol *m*/*z* 376 parent ion. Extracts from wild type VA-MontCo1 accession roots showed similar C15:0d-, C15:1a-, and C15:2a-cardanol fragmentation ion abundances as the extracts from the HR32-4a hairy root line (Table 1). Therefore, the C15:1-cardanols were similar between authentic C15:1-cardanol standard, wild type nascent emerged poison ivy seedlings and hairy roots.

### 2.5. Significant Accession-Level C15-Cardanol Accumulation Levels

Total C:15-cardanol levels were estimated from the shared *m*/*z* 180 fragmentation ion. Figure 1b demonstrates that steady state total C15-cardanol accumulation levels were intermediate between total C15-urushiol and total C17-urushiol accumulation levels. Total C15-cardanol steady state accumulation levels were significantly different across the six poison ivy accessions (two factor ANOVA, whole model r^2^ = 0.75; accession factor F = 2.65, *p*-value = 0.0431). The Tukey HSD analysis did not isolate any significantly different pair-wise least square mean total C15-cardanol differences. However, a contrast comprised of Iowa and New Jersey (with the lowest least square means) as a group vs. the remaining four accessions was significantly different, *p*-value = 0.0243.

**Figure 7:**
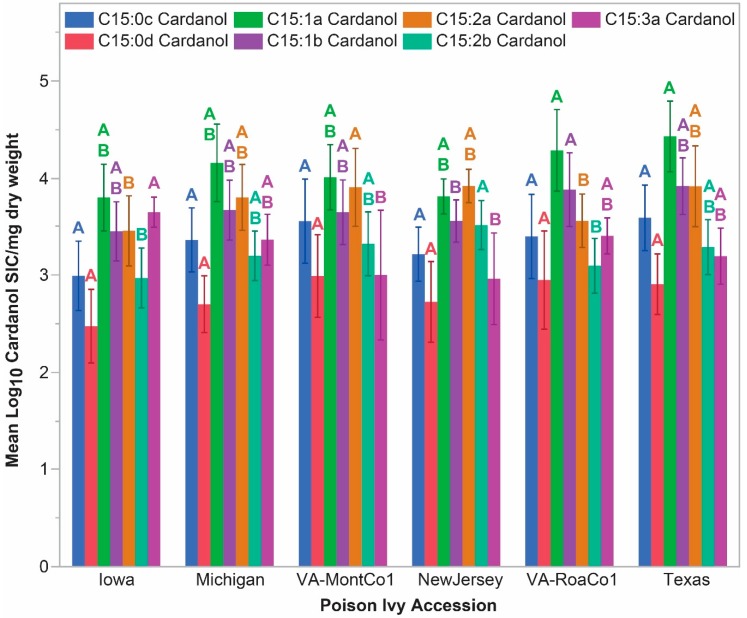
C15-cardanol congener and isomer steady state accumulation levels. Error bars indicate standard deviation. Non-shared letters of the same color indicate statistically significantly different least square means between the poison ivy accessions.

**Figure 8:**
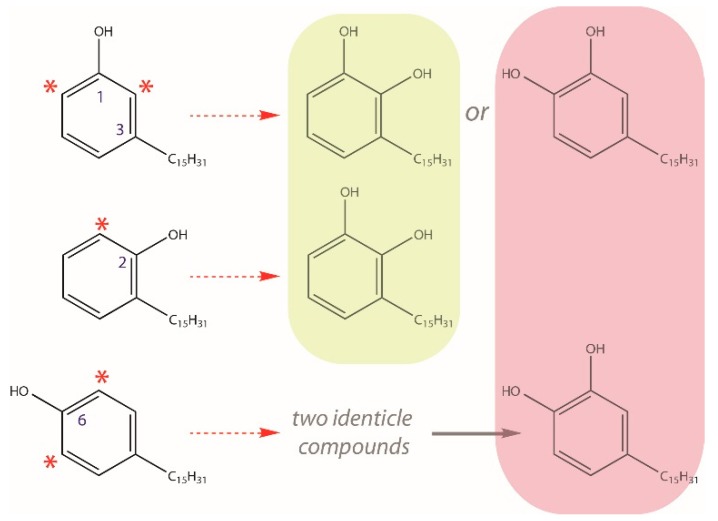
Potential C15:0-cardanol isomer substrates and putative cardanol hydroxylase urushiol reaction products (shaded). Numbers indicate harmonized aromatic carbon positions relative to a stated 3-pentadecyl alkane chain. Red asterisks indicate hydroxyl-proximal aromatic carbon atoms that would be preferentially hydroxylated by a proposed cardanol hydroxylase enzyme activity (red arrows). Proposed urushiol isomers are indicated by different shaded boxes.

Based upon unique elution times, two C15-cardanol isomers were identified for each of the four expected degrees of (un)saturation in the pentadec(en)yl chain. Figure 5a,b shows the relative order of elution of C15-cardanols from the gas chromatography: C15:1a = C15:3a, C15:2a, C15:1b = C15:3b, C15:2b, C15:0d, and C15:0e. Selective ion current (SIC) levels for the C15:3b-cardanol were frequently at or below the limit of detection; therefore, C15:3b levels were not quantified. Four of the seven C15-cardanol congeners/isomers (C15:1b, C15:2a, C15:2b, and C15:3a) showed significant accession-level differences in their steady state accumulation levels (Figure 7). For the same reasons outlined for the C15-urushiol isomers above, structural determination of the C15-cardanol isomers were also not pursued. With that said, C15:0d- and C15:0e-cardanol isomers must have differed only in the relative positions of the hydroxyl vs. the 3-pentadecyl chain on the aromatic ring. For the sake of consistency when referring to the isomeric hydroxyl group positioning on urushiol and cardanols, the position of the pentadec(en)yl chain is always placed at the third carbon of the aromatic ring relative to the 1-hydroxyl group of typical urushiol and cardanol structures [9] (Figure 8). One of the two possible hydroxyl orientations must have been a meta-orientation, because the authentic C15:1-cardanol standard (known meta-orientation of hydroxyl to the 3-pentadecenyl chain) co-eluted with the C15:1a-cardanol isomer (Figure 6). Whether the other C15:1-cardanol isomer was ortho- or para-orientation relative to the 3-pentadecenyl chain was likewise not determined. Searches for C17-cardanol-specific *m*/*z* ions 404, 402, 400, and 398 did not elicit any discernable peaks, presumably because they were below the limit of detection.

## 3. Discussion

Previous reports of poison ivy yielded highly variable estimates of relative C15- and/or C17-urushiol congener accumulation levels. The likely reason for this variability was the ad hoc manner in which the poison ivy plant materials were originally collected, especially with respect to a wide range of biological and environmental variables other than which organs were harvested (e.g., leaf, bark, stem, drupes), location (city and state), and date of harvest. Most studies did not indicate the environmental context that the plants were collected from, nor demographic attributes such as plant age, leaf/branch number, growth habit (climbing vs. ground creeping liana), plant size, soil type, or sex of the harvested plant material. Not surprisingly, other than total C15-urushiol levels being substantially larger than total C17-urushiol levels, reports of relative C15- and C17-urushiol congener levels varied greatly between studies [11,27,28,29,30]. Given that poison ivy shows accession-level differentiation in a number of biometric traits, genotypic differences may have also contributed to the previous reports about urushiol congener accumulation levels. In summary, previous urushiol assessments were highly confounded by genotype, environment, and genotype by environment interactions. An important technical advancement for disentangling environmental contributions from genetic contributions in urushiol accumulation levels is the development of procedures to germinate and grow axenic poison ivy seedling in vitro [24], thereby controlling all of the most salient environmental parameters.

In this study, the genetic composition of poison ivy seedlings was managed by using poison ivy drupes from distant geographical locations (accessions), and in one case a single female liana (VA-RoaCo1). Similar to two previous reports [27,28], across all accessions in this study total C15 levels were substantially greater than total C17 urushiol levels. A variety of poison ivy morphometric traits show significant accession-level differences in a common garden greenhouse experiment [23]. In this study, we extended significant poison ivy accession-level differences to urushiol chemical diversity in total C15-urushiol, total C17-urushiol, ratio of total C15:C17-urushiols, C:15-urushiol congeners, and C17-urushiol congeners steady state accumulation levels. The accession-level differences in urushiol congener steady state accumulation levels demonstrated that presumably chemical defensive traits (i.e., C15- and C17-urushiols) were also significantly differentiated between geographically-isolated poison ivy populations. Given this experimental design in the present study, the manifest differences in urushiol accumulation levels between different accession were due to genetic differentiation between the poison ivy accessions.

The identification of at least two urushiol isomers for each C15-urushiol congener of a given degree of unsaturation was mostly unexpected. Two C15:1-urushiol isomers (C15:1α and C15:1β) [26] and two C17:2-urushiol isomers [27] in poison ivy are previously reported. In this study, four C15:1-urushiol isomers were observed, doubling the number of previous two C15:1-urushiol isomers. Given the difficulty in chemically purifying urushiol congeners structural determination of the urushiol congener isomers was not practical. Nevertheless, several conclusions can be drawn about the structure of a few of the C15-urushiol congener isomers. Because there is no unsaturation in the C15:0-alkane chain, the C15:0d- and C15:0e-urushiol isomers must differ in the relative position of the ortho-hydroxyl groups on the aromatic ring relative to the 3-pentadecyl-chain. The presence of the *m*/*z* 179 ion in the C15:0d and C15:0e-urushiol peaks confirms the presence of the ortho-hydroxyl groups [31], and thus the isomerization cannot be due to meta-hydroxyls on the aromatic ring. This constrains the ortho-dihydroxyl positioning to either 1,2-dihydroxy or 1,6-dihydroxy relative to the 3-pentadecyl group on the benzene ring of the unsaturated C15:0-urushiol isomers. These deductions about the C15:0-urushiol isomer structures were not empirically validated, but derive from first principles. The chemical structures for the two C15:2-and two C15:3-urushiol isomers may be due to the same alternative ortho-dihydroxyl positioning on the aromatic ring, or alternatively could be due to cis/trans orientation at one of the double bonds on the pentadeca(di/tri)enyl chain. The most parsimonious explanation of the four C15:1-urushiol isomers is isomerization of both ortho-hydroxyls positioning on the aromatic ring, combined with cis/trans isomerization at the Δ8 double bond on the 3-pentadecenyl chain. These ortho-dihydroxy-C15-urushiol isomers are especially noteworthy when considering the concomitant identification of C15-cardanol isomers connoted to be the immediate precursors to the C15-urushiol congeners and isomers.

Although not explicitly stated by Dewick [15], C15-cardanols are the implicit decarboxylated metabolites derived from the explicitly proposed C15-anacardic acid intermediate in C15-urushiol biosynthetic pathway. For this reason, trimethylsilane-derivatives of C15-cardanol congeners with predicted parent *m*/*z* ratios of 376, 374, 372, and 370 were queried as selective ions (corresponding to C15:0-, C15:1-, C15:2-, C15:3-cardanols, respectively). These parent *m*/*z* ions eluted as adjacent peaks to one another in the gas chromatograms, and all produced the expected common *m*/*z* 180 fragmentation ion, as well as unique *m*/*z* fragment ions characteristic of C15:1-cardanol congeners ([18], Table 1). Lastly, a predicted C15:1-cardanol peak co-eluted with an authentic C15:1-cardanol standard. To the best of our knowledge, this is the first report of C15-cardanol congeners and their respective isomers in a Toxicodendron species. Cardanols are present in other members of the Anacardiaceae family such as cashew [18]. For the same reasons outlined for the unsaturated C15:0-urushiol isomers, the only potential sites for C15:0-cardanol chemical isomerization was the relative position of the single hydroxyl group to the 3-pentadec(en)yl group on the aromatic ring.

Another line of evidence that specific C15-cardanol congeners are the immediate precursors to their desaturation-matched C15-urushiol congeners was a general (but not absolute) correlation of the number of C15-cardanol congener isomers to the same number of C15-urushiol congener isomers. For example, there were two unique unsaturated C15-cardanol isomers (C15:0c- and C15:0d-cardanol) and two unsaturated C15-urushiol isomers (C15:0d- and C15:0e-urushiols). Likewise, there were two C15:2-cardanol isomers and two C15:2-urushiol isomers; and two C15:3-cardanol isomers with corresponding two C15:3-urushiol isomers. The simplest scenario is that all C15:2- and C15:3-urushiol isomers are similarly isomeric in the positioning of the hydroxyl relative to the 3-pentadic(en)yl group on the aromatic ring, as the C15:0-urushiol isomers. However, it is also formally possible that the isomerization occurs at one of the di- or tri-olefin groups in the unsaturated C15:2- and C15:3-cardanol congeners. The one exception to the parallelism between the number of cardanol and urushiol isomers was two C15:1-cardanol isomers compared to four C15:1-urushiol isomers. This doubling in the number of C15:1-urushiol isomers could be most parsimoniously explained by isomerization about two possible ortho-hydroxyl positions on the aromatic ring combined with cis-trans isomerization about the Δ8 double bond on the 3-pentadecenyl chain.

A generalized model for the penultimate step for urushiol biosynthesis in poison ivy newly-emerged seedlings is illustrated in Figure 8 and outlined as follows. Two underlying assumptions are made in this biosynthetic model. The first assumption is that all C15-urushiols are isomers differing in the relative position of the ortho-dihydroxy groups to the fixed 3-pentadec(en)yl-group. Only two C15-urushol isomers fit this criterion: 1,2-dihydroxy-3-pentadec(en)yl-benzene and 1,6-dihydroxy-3-pentadec(en)yl-benzene. The second assumption is that these C15-urushiol isomers are derived from as many as three potential C15-cardanol isomers: 1-hydroxy,3-pentadec(en)yl-benzene, 2-hydroxy,3-pentadec(en)yl-benzene, or 6-hydroxy,3-pentadec(en)yl-benzene by a proposed cardanol hydroxylase activity that preferentially adds a hydroxyl group adjacent to the pre-existing single hydroxyl group on the aromatic ring of theC15-cardanol molecule.

The first assumption that all C15-cardanols are isomeric in the position of the hydroxyl group relative to the 3-pentadec(en)yl chain currently appears to be a special case of nascent-emerged poison ivy seedlings (comprised of largely embryonic-derived tissues). True leaves collected from wild poison ivy plants do not show multiple isomers of C15:0-, C15:2-, and C15:3-urushiols [11,26,27,28,29]. The nascent-emerged poison ivy seedlings were harvested as intact seedlings, so it is formally possible that either: embryonic organs differentially-produced particular cardanol/urushiol isomers, or all embryonic-derived organs produced and accumulated all cardanol/urushiol isomers.

The identification of steady state C15-cardanol accumulation levels in poison ivy seedlings provided empirical evidence supporting the hypothesis that C15-cardanols are the penultimate metabolites in C15-urushiol biosynthesis. The proposed cardanol hydroxylase enzyme activity provides several testable hypotheses to advance future detailed studies of C15-urushiol biosynthesis. Another notable observation was the overall positive correlation of nearly all C15-cardanol, C15-urushiol, and C17-urushiol congener steady state accumulation levels with each other. Supplemental Table S1 shows the Pearson correlation coefficients and their associated *p*-values for all pairwise comparisons of all C15-cardanols, C15-urushiols, and C17-urushiols. 273 out of 276 pairwise metabolite comparisons showed moderate to high Pearson coefficient values with statistically significant support (*p*-values < 0.05). The positive strong correlation of C15-cardanols with C15-urushiols is consistent with C15-cardanols serving as the metabolic precursors to C15-urushiols. However, C15-urushiols are not believed to be the metabolic precursors to C17-urushiols, because each are most likely derived from different fatty-acid pools (i.e., C16-fatty acids and C18-fatty acids, respectively). Therefore, the statistically significant correlation of C15-urushiol and C17-urushiol congener accumulation levels is perhaps due to one or more shared-regulatory mechanisms acting on both C15- and C17-urushiol biosynthetic genes. Moreover, coordinate regulation of C15- and C17-urushiol biosynthetic genes is likely not occurring in the same cells, because 2D-MALDI high resolution MS analyses indicate that C15-urushiols accumulate in different cells than C17-urushiols in poison ivy stems [25].

## 4. Materials and Methods

### 4.1. Poison Ivy Seedling Germination and Harvest

Poison ivy drupes used in this study were collected from four previously described sites in the USA [23], as well as one site in New Jersey (39°54′14″ N 74°54′4″ W) and an additional site in Virginia (VA-MontCo1, 37°13′42.79″ N 80°25′58.56″ W). Ten axenic poison ivy drupes from each location were germinated in a randomized design to yield axenic seedlings [24] that grew in the light until the seedling stood upright and one or both of the cotyledons fully released from the endocarp. Within 24 h of endocarp release each seedling was harvested intact, placed into a pre-weighed 1.5 mL microfuge tube, flash frozen in liquid nitrogen, ground to a powder with a microfuge tube-fitting mortar, and then stored at −80 °C. One day prior to urushiol extraction, the frozen microfuge tube cap was removed, and the cap was replaced with a modified microfuge cap into which a 200 µL filter tip was inserted. The modified microfuge tubes containing frozen homogenized poison ivy seedling material were lyophilized to remove all water from the samples. Upon removal from the lyophilizer, the original microfuge cap was reinserted and the microfuge tube mass was measured using a Mettler Toledo (Columbus, OH, USA) XS205 Dual Range analytical balance. Based on the net microfuge tube weight and the empty microfuge tube tare weight, the dry biomass of the poison ivy seedling was calculated. Poison ivy hairy root line HR32-4a was established by prick inoculating a poison ivy hypocotyl with *Agrobacterium rhizogenes* strain ATCC15834, resulting in a hormone-independent poison ivy hairy root culture (manuscript currently under review, or unpublished data, J. Jelesko).

### 4.2. Alkylphenol Extraction and Assay

Alkylphenols were extracted and quantified using a previously established trimethylsilane-derivatization and GC-MS detection protocol, using the same instrument and chemicals as extensively described in [25]. Given the total number of poison ivy seeding samples and the number of samples that could be accommodated by the nitrogen evaporative manifold, the alkylphenol extraction and GC-MS analysis procedure was performed in batches of three to four samples of two to five poison ivy accessions per batch over the course of several days to complete alkylphenol estimation levels. Urushiol and cardanol congeners were identified based on published TMS-derivative parent and fragmentation *m*/*z* ratios [18,31] listed in Table 1. Steady state alkylphenol levels were reported as selective ion counts (SIC) (normalized with sample extraction efficiency based on internal standard recovery) divided by the sample dry weight (i.e., normalized selected ion count (SIC)/mg dry weight). If more than one urushiol or cardanol isomer were observed in a chromatogram, the first eluting isomer was labeled with a lower-case letter “a” suffix, and subsequent isomers with serial letter suffix designations. Presumed M+2 artefactual congeners were additionally labeled with an asterisk (*). Total C15- and C17-urushiol levels were estimated using the common *m*/*z* 179 fragmentation ion [31]. Total cardanol levels were integrated using the cardanol common *m*/*z* = 180 fragmentation ion [18]. Authentic C15:1-Cardanol (3-8Z-pentadecen-1-yl-phenol, catalog # 23154) was obtained from Cayman Chemical (Ann Arbor, MI, USA).

### 4.3. Statistical Analysis of Urushiol and Cardanol Congener Steady State Levels

The program JMP Pro v14 (SAS Institute, Cary, NC, USA) was used to perform the parametric linear regression modeling of the urushiol and cardanol (alkylphenol) accumulation data. Steady state alkylphenol congener dependent variables were Log_10_ transformed to achieve a reasonable fit to a Gaussian distribution as evaluated by box plot and normal quantile plots. Optimal ANOVA linear models were determined beginning with complete models comprised of alkylphenol analysis batch date and poison ivy accession as independent categorical factors (fixed effects) and their interaction term, followed by subsequently simpler models attained by sequentially removing one factor at a time. The final optimal model was determined by identifying the linear regression model with the lowest AICc value, thereby identifying the optimal model fit to the data. In all but a few dependent variable models, the optimal model was comprised of “accession” and “alkylphenol analysis batch date” as the significant predictor/independent factors. Pairwise comparison of the least square mean estimates for various poison ivy accession treatment levels was performed using the Tukey HSD analysis.

## 5. Conclusions

Poison ivy seedlings showed significant accession-level differences in steady state urushiol congener accumulation levels, consistent with underlying genetic differentiation between geographically isolated poison ivy populations. The seedlings also revealed novel C15-urushiol isomers of all four urushiol congeners differing in their degree of unsaturation in the pentadec(en)yl chain. In the case of the C15:0 urushiol isomers, the structural differences must have been due to differential positioning of the two ortho-hydroxyls relative to the pentadecyl chain on the aromatic ring. Metabolite profiling also revealed cardanol congeners with four different degrees of unsaturation in the pentadec(en)yl chain that likewise showed isomeric forms. The cardanols also showed statistically significant accession-level steady state accumulation levels. The parallels between C15-cardanol congener structures and isomeric forms with analogous C15-urushiol congeners and isomers led to the hypothesis that a cardanol hydroxylase enzyme activity is responsible for converting the penultimate metabolite cardanol into the ultimate product urushiol. The identification of cardanols in poison ivy provide the first empirical evidence for a previously hypothesized/implied metabolic intermediate in urushiol biosynthesis.

## Figures and Tables

**Table 1:** Trimethyl-silane (TMS)-derivatized C15-cardanol congener parental and fragmentation *m*/*z* ratios. Bracketed text indicates actual congener. Underlined indicates parent ion *m*/*z*. Bold indicates common cardanol fragmentation ion *m*/*z* 180. Parentheses bound the percent abundance relative to the most abundant fragmentation *m*/*z* 180 ion.

Sample:	TMS-Derivatized Cardanol Parent and Fragmentation Ions:			
	C15:0	C15:1	C15:2	C15:0d
Cardanols isolated from Cashew [18]	376 (77) 361 (5) **180** (100) 179 (25)	374 (42) 359 (2) **180** (100) 179 (22)	372 (34) 357 (4) **180** (100) 179 (39)	376 (77) 361 (5) **180** (100) 179 (25)
HR32-4a	[C15:0a*] 376 (3) 361 (0) **180** (100) 179 (39)	374 (23) 359 (2) **180** (100) 179 (39)	Substantial overlap with C15:1a.	B.L.D.
C15:1 cardanol authentic standard	[C15:0a*] 376 (2) 361 (0) **180** (100) 179 (31)	374 (25) 359 (2) **180** (100) 179 (31)	N.A.	N.A.
HR32-4a with 1ng authentic C15:1 cardanol spike	[C15:0a*] 376 (2) 361 (0) **180** (100) 179 (28)	374 (24) 359 (2) **180** (100) 179 (28)	Substantial overlap with C15:1a.	B.L.D.
VA-MontCo-1 Seedling	[C15:0a*] 376 (2) 361 (0) **180** (100) 179 (27)	374 (20) 359 (1) **180** (100) 179 (27)	372 (14) 357 (2) **180** (100) 179 (40)	376 (30) 361 (2) **180** (100) 179 (31)

## Supplementary Materials

The following are available online, Figure S1. Selective ion chromatograms of C15- and C17-urushiol congeners and isomers. Table S1. Pearson correlation results for all C15-cardanol, C15-urushiol, and C17-urushiol congener and isomer pairs.
